# What is morally at stake when using algorithms to make medical diagnoses? Expanding the discussion beyond risks and harms

**DOI:** 10.1007/s11017-021-09553-0

**Published:** 2022-01-01

**Authors:** Bas de Boer, Olya Kudina

**Affiliations:** 1 University of Twente, Enschede, Netherlands; 2 Technische Universiteit Delft, Delft, Netherlands

**Keywords:** Algorithms, Machine learning, Ethics, Medical diagnosis, Technomoral change, Technological mediation

## Abstract

In this paper, we examine the qualitative moral impact of machine learning-based clinical decision support systems in the process of medical diagnosis. To date, discussions about machine learning in this context have focused on problems that can be measured and assessed quantitatively, such as by estimating the extent of potential harm or calculating incurred risks. We maintain that such discussions neglect the qualitative moral impact of these technologies. Drawing on the philosophical approaches of technomoral change and technological mediation theory, which explore the interplay between technologies and morality, we present an analysis of concerns related to the adoption of machine learning-aided medical diagnosis. We analyze anticipated moral issues that machine learning systems pose for different stakeholders, such as bias and opacity in the way that models are trained to produce diagnoses, changes to how health care providers, patients, and developers understand their roles and professions, and challenges to existing forms of medical legislation. Albeit preliminary in nature, the insights offered by the technomoral change and the technological mediation approaches expand and enrich the current discussion about machine learning in diagnostic practices, bringing distinct and currently underexplored areas of concern to the forefront. These insights can contribute to a more encompassing and better informed decision-making process when adapting machine learning techniques to medical diagnosis, while acknowledging the interests of multiple stakeholders and the active role that technologies play in generating, perpetuating, and modifying ethical concerns in health care.

## Introduction

Machine learning techniques are increasingly used in medical diagnostic practices, bearing the promise of a more efficient medical diagnosis process with a significantly lower margin of error. At present, clinicians and care teams already use machine learning (ML) systems, especially in the context of diagnostic imaging, for improving early detection of melanoma and sepsis, detecting cardiac arrythmia, ischemia, and retinopathy, predicting breast cancer development based on node biopsy results, and choosing between competing diagnoses [1]. ML is celebrated for reducing the supposedly subjective aspects of diagnosis by medical practitioners and for increasing the trustworthiness of the diagnoses given insofar as it enables medical judgments that purportedly rely on the objective outcome of algorithmic processes [2–4]. In this way, so it is argued, ML may help to eliminate the perceptive bias inherent to the sensory capabilities of human beings and the forms of contextual bias that may in the relation between patients and health care providers. It is thought that these factors, which are perceived as unwarranted subjective interferences in otherwise objective diagnostic processes, are neutralized when relying on algorithms that are immune to such intrinsic or contextual biases.

However, some argue that overreliance on ML could have unintended negative consequences. They fear that ML algorithms will introduce a situation in which neither medical practitioners nor patients understand why a certain medical diagnosis is made, thereby reducing the potential for critical reflection [5]. Furthermore, ML models are dependent on the training data fed into the algorithms that build them, which makes their decisions dependent on what information is considered valuable by the individuals responsible for generating the algorithms’ input [6]. In other words, medical diagnoses obtained using ML models are simultaneously value-laden and opaque. Although it is in principle possible to detect which values have been fed into an ML algorithm, there is no certainty about how these values inform the diagnosis output using the model that the algorithm has trained on their basis.

Ethical analyses of the use of ML in medical diagnosis typically focus on technological, quantifiable concerns related to risk, efficiency, and safety [7]. This focus on the quantifiable is reflected in the literature on this subject, which is typically concerned with objective and measurable impacts of ML on the safety of patients—for example, whether or not ML increases the likelihood of misdiagnosis [2, 3, 8, 9]. Placing too much emphasis on such *hard impacts* of medical technologies prevents due consideration of their *soft impacts* [10]. The soft impacts of technologies are their qualitative moral effects, such as changes in the extent that doctors can be held responsible for diagnostic outcomes, in the relationship between patients and medical professionals [11], or more generally in the standards for what constitutes good health care practices [12].

In this paper, we present several possible soft impacts of the use of ML in medical diagnosis. To do so, we draw on two approaches that attempt to trace and conceptualize the qualitative dimensions of the introduction of new and emerging science and technologies: *technomoral change* (TMC) and *technological mediation theory* (TMT). Both are concerned with investigating how technologies co-shape how the world appears to individuals and how they alter moral frameworks. These two (competing) approaches show a close resemblance, which makes it surprising that a comparative discussion between them is thus far absent from the literature. In this paper, we will compare the approaches by applying them to analyze ML in medical diagnosis.

The paper is structured as follows: We first describe the approaches of technomoral change and technological mediation, their assumptions and starting points, and elaborate on the similarities and differences between the two. We then examine the use of ML as a form of applied artificial intelligence (AI) in medical practice through the lenses of TMC and TMT and analyze the epistemic, existential, and legal concerns that they elucidate. Third, we argue that both approaches can help to highlight the relevance of the qualitative moral impact of ML against the dominant discourse on quantitative measurements of risk and safety in ethical discussions about ML. To conclude, we reflect on how a combination of TMC and TMT contributes to participatory engagement in the context of medicine and expands the current scope of ethical conversation about the role of ML in medical diagnosis.

## Accounting for the qualitative moral impacts of technologies: technomoral change and technological mediation

The use of ML to improve medical diagnoses should not be considered a neutral aid to existing forms of observing and reasoning about physiological anomalies. Rather, new technologies—such as ML—have a profound ethical dimension. By embodying certain values, technologies can inspire specific courses of actions and ways of understanding the world [13], foster particular forms of moral engagement and allow people to form new relations with their surroundings [14], and provide individuals with new moral insights and intuitions [15]. For example, Ciano Aydin suggests that “what is considered ‘normal’ and ‘healthy’ is redefined in terms of what technologies are able to measure, diagnose, and treat” [16, p. 318]. In other words, technologies reshape the meaning of medical concepts, as well as how these concepts are applied in clinical practice. TMC and TMT are two approaches advanced in the philosophy of technology that take these insights into account by treating morality as a hybrid affair involving people and technologies [17, 18].

### Technomoral change

The approach of TMC holds that people’s normative frameworks are not static but co-evolve with the introduction of new technoscientific developments [17]. Drawing on John Dewey’s pragmatism, it suggests that new technoscientific developments serve as moral provocateurs, robbing the usually dormant moral norms of their self-evident nature, destabilizing tacit values, and opening moral routines up to critical reflection [19]. Consequently, they may effect a shift in the values brought to bear in a given context and the ways these are seen to translate to moral action. For instance, in the 1960s the value of care toward others and good manners might have dictated offering a cigarette to one’s guest. Nowadays, such an offer might be considered inappropriate (without prior knowledge of a guest’s smoking habits), since smoking does not accord with the norm of what constitutes a healthy lifestyle and inviting a guest to partake as such presents an antithetical manifestation of what care means [20]. Overall, TMC is interested in elucidating and explaining the relation between technologies and moral change.

TMC emerged to facilitate ethical decision-making—primarily at the policy level—pertaining to new and emerging technologies [20, 21]. Acknowledging the intricate interplay between values and technologies (conceived as moral disruptors), it becomes desirable to identify and reflect on the specific potential moral impacts of new technologies. To this end, TMC aims to fill a lacuna in policymaking by focusing on the soft impacts of technologies. Traditionally, policy discussions have concerned the measurable, traceable, and visible technological risks, costs, and benefits. Tsjalling Swierstra and Hedwig te Molder argue that the subtle qualitative effects of technologies need to be included in decision-making as well [10]. Although they are more difficult to pinpoint and account for, soft impacts weave into the canvas of human lives as technologies continually work to expose moral standards that no longer work, define new norms, and shape infrastructural and institutional processes. Soft impacts of technologies, in short, are no less important than risk and cost–benefit analyses.

### Technological mediation theory

TMT combines empirical and philosophical analysis to reflect on people’s current or anticipated interactions with new technologies, as well as on the myriad normative, epistemic, and existential concerns that such interactions may give rise to [22–24]. Given its phenomenological origins, TMT primarily uses case studies to analyze human–technology relations at the micro-level, while maintaining a rigorous philosophical perspective on how technologies help shaping people’s moral views, preferences, choices, and actions.

TMT considers technologies to be mediators of human–world relations [18]. As mediators, technologies “co-shape, enable, challenge or change the engagement of people with the world” [22, p. 302], thereby actively shaping human experiences, practices, and value frameworks. Overall, the aim of TMT is to examine how people relate to themselves and to the world around them in view of specific technologies—looking at the quality and structure of such relations [18, pp. 15–16].

By co-shaping moral actions and decisions, technologies mediate morality: prenatal genetic testing mediates moral questions about childbearing, semiautonomous robots mediate moral experiences of war, and closed-circuit television cameras mediate moral standards of behavior in public places. As Olya Kudina and Peter-Paul Verbeek note, “If ethics is about the question of ‘how to act’ and ‘how to live,’ and technologies help to shape our actions and the ways we live our lives, then technologies are ‘actively’ taking part in ethics” [25, p. 297].

Recently, proponents of TMT have argued that technologies not only co-shape situations of moral choice, but also mediate the infrastructure of morality—the meaning of human values [25, 26]. Technologies introduce new courses of action and open up new moral avenues, thereby inviting existing values to be revisited or reaffirmed [18, 27]. TMT can capture such value dynamism by conducting joint empirical and philosophical analyses of how individuals engage with or foresee engaging with technologies. Such joint analyses make space for exploring the impact of technologies on individuals’ daily lives and into the concerns that they might have or anticipate having. Thus, through TMT one is able to probe how specific forms of moral understanding are being (re)invented in relation to technologies or, put differently, to uncover the moral hermeneutics of technologies. By making existing and foreseen moral mediations explicit, TMT enables individuals to think about their interactions with technologies in an informed way.

### Theoretical differences between TMC and TMT

Although the technomoral change approach and technological mediation theory both attend to the mutual interplay between values and technologies, they do so toward different ends and with different emphases. On the one hand, TMC aims to deepen and substantiate policymaking discussions regarding the future of a given technology in society [17, 19, 28]. To that end, it uses a broad societal lens to explore how technologies change values at the infrastructural and institutional levels, as composite renderings of accumulated individual changes. On the other hand, TMT focuses more on the individual level to inform the practices of technological design and use [29, 30], standing on the shoulders of (post)phenomenology with a commitment to lived experiences and the first-person perspective. To that end, it explores the moral mediation of new technologies through the lens of specific human actors, looking at how people appropriate new technologies and make them meaningful and how technologies mediate their concrete experiences and practices. TMC does not necessarily exclude such an individual viewpoint; rather, its interest in galvanizing group ethical deliberations calls for analyses that are scaled up from particular experiences in order to address broader, generalized concerns that many people can relate to. Similarly, by cultivating a new focus on how values change as a result of technological developments, TMT can potentially extend beyond individual concerns and lend itself to discussions at a larger scale, informing group ethical deliberations [31].

Another point of distinction between the approaches concerns their scope and object of interest. Whereas TMC looks at value change over time, TMT explores how technologies mediate values in the here and now. TMC considers change in values over a broad temporal trajectory [17], while TMT zooms in on lived practices to show how different dimensions of values materialize in the present [25]. From this temporal perspective, it might be said that the scope of TMC is larger than that of TMT, the latter of which scrutinizes technologically mediated value dynamism as it occurs in human–technology encounters. Nonetheless, the somewhat narrower focus of TMT allows it to expose and expound the dynamics of value change itself, something that currently lacks sufficient treatment in TMC.

These distinctions between TMC and TMT are not clear-cut as the above discussion suggests, and both approaches can be seen to mutually inform one another insofar as they represent different aspects of the same phenomena. There would be no technomoral change without technologically mediated value dynamism; and value dynamism, however foundational, is a first step in the larger process of value change. While the intent and scope of TMC and TMT are roughly sketched above, we have not yet delineated how combining these approaches would look in practice. Assessing their practical utility in a more substantial way requires paying attention to the methods of analysis that they respectively endorse. We now turn to the case of ML in medical practice to scope out what each of the two approaches can contribute to understanding the technology’s ethical significance.

## The moral impact of using machine learning in medical diagnosis: epistemic, existential, and legal concerns

In the context of medical diagnosis, ML learning algorithms are used to train models on the basis of sample data about particular cases. On the basis of these models, predictive algorithms are developed that render diagnoses. Using ML in medical practice affects the observation capacities of medical professionals in the process of diagnosis. Through the presence of ML, medical professionals, patients, and the relationships between them are co-constituted in new ways. To explore the moral impact of such co-constitutions, in this section we analyze three umbrella categories of concerns raised in discussions, largely among medical specialists and computer scientists, about the potential ethical problems that might arise when introducing ML and AI systems into medical practice: (1) epistemic, (2) existential, and (3) legal. Through the lenses of TMC and TMT, we interpret what are often presented as technical problems within these categories as *moral* problems, in that they also represent changes in the norms and values that constitute good medical practice. In this way, rather than present the concerns raised about ML as specific issues that need solving (e.g., risk of misdiagnosis or construal of patients as sets of data points), we elucidate the ways in which ML might give rise to new norms, such as by co-shaping how health professionals acquire medical knowledge, how the responsibility of medical practitioners is interpreted, or how meaningful doctor-patient relationships are formed (see [11]).

The categories we employ can be loosely characterized as follows: *epistemic* concerns are those about how ML changes the knowledge needed to arrive at a diagnosis; *existential* concerns are those related to how ML changes the way that relevant stakeholders (i.e., medical professionals, patients, and developers) understand themselves and each other; and *legal* concerns are those regarding the foreseen challenges that ML poses for existing forms of legislation. In practice, these categories cannot be so neatly separated because the soft moral impacts of technologies often flow from one aspect of medical practice into another—changes in the epistemology of diagnosis might alter how doctors and patients understand their responsibilities, which in turn might lead to reform of existing legal regulations. However, for our purposes, organizing concerns in this way is useful for delineating the different aspects of medical practice that might be reshaped by the introduction of ML in medical diagnosis.

### Epistemic concerns

The first set of concerns that we focus on are epistemic ones, dealing with how relevant knowledge for coming to a diagnosis should be obtained. We focus on two main epistemic concerns in the literature, namely biases that can be present in ML, and the opacity of ML.

#### Human bias and machine learning bias

The medical literature often discusses ML in terms of its potential to eliminate subjective biases. Subjectivity as such is treated as an epistemic problem characterized by the presence of human bias—a human factor that intervenes unwantedly in otherwise objective processes and increases the likelihood of diagnostic errors [2–4, 9]. On such a view, medical diagnoses are objective judgments that classify a given state or set of states as indicative of the presence of a specific disease or condition, suggesting a course of action that allows this disease or condition to be treated or mitigated accordingly. Since medical practice invariably involves human subjects, an important concern is how these human factors influence diagnostic processes. When ML is approached from this perspective, one of the central concerns is the objectivity of medical knowledge generated in relation with ML algorithms. The main questions that arise, then, are about which forms of bias might be (re-)introduced by ML models and how such biases can be eliminated [5, 8, 32].

Training ML models to aid in medical diagnosis requires an enormous amount of sample data drawn from particular cases. Hence the performance of these models is contingent on hospitals’ willingness to donate generous volumes of their patients’ data to the databases of companies that own ML algorithms [4, 33]. Crucially, the data fed into the ML algorithm must not overrepresent specific types of patients, because “biases in data collection can substantially affect both performance and generalizability” [4, p. 1217].

The example of IBM’s attempted diagnostic application of Watson, one of the most frequently used intelligent decision support systems, illustrates the importance of considering what data are fed into ML algorithms. In launching Watson for Oncology, IBM’s ML model was trained on data gathered from a single cancer-treating hospital in New York, serving generally affluent patients with relatively homogeneous social and cultural backgrounds [34]. As a result, the treatment options offered by the Watson for Oncology platform favor specific approaches to health care over others. For example, oncologists in Taiwan observed that Watson’s therapeutic recommendations exhibited bias toward the way drugs are prescribed in the United States, failing to take into account that compared to American patients, Taiwanese patients “often receive lower doses of drugs to minimize side effects” [34]. The suggestions made by the system therefore required critical interpretation by Taiwanese oncologists, failing to deliver the projected time and cost reductions. Yet insofar as ML models come to be regarded as objective sources of knowledge, physicians’ confidence to engage in such critical interpretation, or otherwise disagree with the treatments suggested by these models, may be undermined [5, p. 517].

With the approaches of TMC and TMT in mind, it becomes clear that data bias is not an isolated epistemic problem, but rather a circumstance that bears on medical practice more broadly. It has a soft impact on hospitals in that attempting to reduce this bias calls for the development of institutional policies that ensure patients’ consent to donate their data, enabling large databases of cases to be compiled from diverse patient groups across different countries. Furthermore, ML mediates the responsibility of health care providers. Since ML models may not be generalizable across contexts and cultures, medical professionals need to be able to critically evaluate the diagnoses and treatments rendered by ML systems. In this way, successful use of ML in medical diagnosis requires that health professionals have sufficient experience or specialized knowledge to fulfill their role as responsible actors.

#### Opacity of algorithms

Regardless of whether the technical means are available to eliminate forms of bias present in ML models, the opaque workings of ML often make it unclear to the involved parties how a specific ML-informed diagnosis is reached [5, 7, 35]. This presents another epistemic concern—that the opacity of ML, their “black box” nature, is likely to limit or even “impede the uptake of these tools into practice” [36, p. 32]. In this regard, it is doubtful that ML will eliminate doctors from the process of medical diagnosis any time soon; at most, ML should be considered an augmentation to diagnosis. Yet even if ML is introduced only to augment current diagnostic practices, the opacity of its operations remains an issue, since its output will still influence diagnoses by suggesting specific ways to interpret a physiological state of affairs, thereby affecting the course of action that medical professionals take [37, p. 42].

Building on the framework of TMT, we propose that the opacity of ML mediates the sense in which health care providers can be considered epistemically responsible agents [38]. If medical professionals provide a diagnosis, they are expected to be able to articulate the process of reasoning by which their diagnosis is derived. To serve as responsible epistemic agents, they should be able to trace the various steps of their diagnostic process (or at least be able to reconstruct their process post hoc). However, because ML makes certain steps leading to diagnosis inaccessible—due to algorithmic opacity—a new situation emerges. How does this situation shape the sense in which doctors are epistemically responsible?

There are two ways in which the opacity of ML can be understood: it may be seen as *relatively* opaque, being opaque to specific users (e.g., doctors) but not to others (e.g., algorithm developers); or as *inherently* opaque, being epistemically inaccessible in principle by virtue of its specific constitution. We will discuss these conceptualizations in turn.

The idea that ML has relative opacity rests on the assumption that its workings can in principle become transparent when the user has been sufficiently informed. For example, algorithm developers may have the knowledge and skills necessary for understanding how ML operates, while doctors and other health professionals may not.

One way to address relative opacity is to point to the normative role of ML development teams. For example, Felicitas Kraemer and colleagues suggest that software designers have a responsibility to apprise users of how their programs operate and why their algorithms are likely to draw certain inferences rather than others [6]. The process of explaining how ML models are trained to predict diagnoses is likely to be hampered by “the fact that the software designer may lack the proper medical background of a fully trained physician” [6, p. 259]. However, it is thought that medical professionals in principle have the ability to become informed users of ML and thereby reduce or eliminate the opacity they are faced with. In this vein, Danton Char and colleagues point to the danger of physicians’ “remaining ignorant about the construction of machine-learning systems” [8, p. 983]. From the perspective of TMT, one can consider ML systems to be a technological mediation of diagnostic practice because they reconstitute what it means to be an epistemically responsible health care provider—specifically, they require providers to take responsibility for the elimination of relative opacity by having sufficient understanding of ML to allow them to link medical knowledge to the workings of algorithms. Furthermore, algorithm developers become agents within medical practice insofar as they have an obligation to be maximally transparent about how ML models are built in order to enable doctors to serve as epistemically responsible agents in the first place.

Alternatively, it might be thought that the opacity of ML is not relative to individual users, but inherent to the technology itself [5, 7, 8, 36, 39]. In that sense, the workings of ML algorithms and models—for example, the procedures leading to the output of a specific diagnosis—are accessible neither to users nor to algorithm developers. This issue is becoming increasingly important: techniques such as neural networks and deep learning reportedly allow for the development of models with a better performance rate, yet make for irretraceable algorithmic operations [40], thus rendering the workings of ML systems using these techniques inaccessible in principle. A white paper by the Canadian Association of Radiologists argues that the fact that the workings of such systems are epistemically inaccessible in principle makes it necessary to “clarify [the] scope of medico-legal responsibilities of AI-supported clinical decisions” [39, p. 131]. Furthermore, Char and colleagues warn that “ethically problematic outcomes” can be expected when ML systems are allowed “to be constructed as black boxes” [8, p. 983]. Such fears run counter to the general optimistic discourse surrounding ML, which celebrates its potential to make the diagnostic process more accurate, relieve doctors of the epistemic burdens of uncertainty, and provide patients with more effective care [4].

Some proponents of using AI in medicine have countered that the opacity of ML systems is equivalent to that of human health care providers and that insistence on explainable ML means foregoing its current accuracy: “When the demand for explanations of how interventions work is elevated above careful, empirical validation, patients suffer, resources are wasted, and progress is delayed” [41, p. 18]. In this regard, the prospect of inherent opacity presents a tough choice: either potentially promising techniques should be discarded from medical practice or an agent should be introduced into medical diagnosis that has a significant influence on diagnostic outcomes but the nature of whose influence remains obscure. In the latter case, it is important that doctors are capable of developing a critical perspective on diagnoses made by ML systems to avoid overreliance on a system that is epistemically nontransparent. Yet the question of how responsibility is distributed across agents in ML-aided diagnosis remains open and points to the need for new legal norms in the regulation of diagnostic practice.

### Existential concerns

The second set of concerns we examine pertain to the way that relevant stakeholders in medical diagnosis experience and understand themselves in relation to their sociomaterial environment. These concerns involve anticipated changes that ML may engender for *medical professionals* and *patients* as the key actors who will use the technology, as well as *developers* as facilitators of the technology in medical practice (who also receive frequent mention in the literature). We refer to these concerns as existential to highlight how ML might shape the role and self-understanding of medical professionals and patients, along with how it might place a responsibility on ML developers to create adequate medical and technological infrastructural support systems.

#### The responsibility of health care providers

The dominant concerns that ML raises for health care providers involve anticipated changes to the meaning and role of health professionals, changes to health care duties and responsibilities, and the fear of overreliance on ML systems. ML is marketed as increasing efficiency and decreasing costs in medical diagnosis, with some even suggesting that “AI will eventually … outperform physicians” [42, p. 391]. While ML systems are not likely to replace doctors in the near future, they already do change how they diagnose. ML has become an active part of medical decision-making; it presents actions and judgments that shape the perceptions of health professionals in a more direct way than other existing technologies, making it more difficult for them to disagree with the interpretations rendered by the system or justify decisions to disregard them, while engendering hesitance to provide independent diagnoses and causing them to distrust their own judgment [5, p. 517].

Health care professionals’ trust in ML, as supported by increasing evidence that they do indeed defer certain decisions to ML systems, may lead to an overreliance on these systems in medical decision-making. The anticipated moral impacts of such overreliance focus on declining awareness of contextual factors not represented in ML training data and on loss of skill in bedside manner, leading to “reduced interest in and decreased ability to perform holistic evaluations of patients” [5] and to a cold style of care in which patients are devalued [11, 36, 43].

Contrarily, it has been proposed that ML could instead contribute to rebuilding empathy and trust in provider–patient relationships in view of its anticipated time-liberating function for health professionals. The argument here is that if properly trained, ML systems can make medical practice “human again,” affording health care professionals more opportunities to exercise compassion and allowing them to be more available for their patients [44]. Such cautious hopes center on the assumption that relieving health care professionals of certain time-consuming duties would translate to their having more time to connect with their patients. Insights from the field of science and technology studies, however, indicate that while the introduction of new technologies may absolve some old obligations, it can also impose new ones, resulting in increased workload, expanded duties, and decreased overall time to carry these out [45]. Similarly, if the emergence of electronic health record systems is any guide, in developing ML diagnostic systems to maximize efficiency and reduce costs of care, the potential increase in providers’ purported “free” time will not automatically result in better care, but rather may lead to feelings of “angst, depression, and disenfranchisement” [46].

Finally, the introduction of ML may change health care professionals’ duties and responsibilities by delimiting them differently and extending them into other domains. For example, the responsibility to give good medical diagnoses is complicated by the inherent opacity of ML systems, requiring health care professionals to understand and evaluate the output suggestions [43]. Additionally, the duty of confidentiality may at times come into conflict with the duty to warn, such as when a patient is at risk of spreading a communicable disease or otherwise endangering identifiable persons. ML systems that support continual learning, in which models evolve adaptively in response to new patient data, can allow for “a broader range of inferences about whether a user poses danger to others” [42, p. 395]. Though not yet codified as a legal duty, this affordance might place a moral responsibility on health care professionals to disclose any information about such potential harm. However, the heightened duty to warn may impair the patient’s trust in health professionals and complicate their relationship with patients. These duties also require health care professionals to have a nuanced understanding of what it is they are warning about—recognizing conflicts of interest between hospitals and the companies behind ML systems, appreciating the complexities of data management and ensuring informed consent to use patients’ medical information, and most importantly, having a solid grasp on how the recommendations of ML systems are produced. Thus, while ML systems might cut down on some aspects contributing to the cognitive load of medical professionals, they might also impose additional burdens, with health care professionals now required to have the ability to understand the algorithmic steps that are applied in the transformation of data into suggestions by ML systems. Health care professionals may derive a sense of support from ML systems, but the newly expressed duty of explainability may equally elicit anxiety and pressure to justify medical choices and ensure informed consent.

Reflecting on the existential concerns raised by ML from the perspectives of TMT and TMC makes clear how this technology could subtly influence how diagnostic practices are organized. For instance, ML systems mediate the role and meaning of the medical profession, implicitly changing daily routines and inviting delegation of certain duties while shifting their perceived importance. A potential soft impact of this mediation is an overreliance on ML in health care which changes the nature of medical practice, as well as a revision (or evolution) of the duties and responsibilities of health care professionals. While inviting them to delegate seemingly benign, repetitive, and time-consuming tasks, ML systems also mediate their self-confidence and willingness to provide conclusive judgments without these technologies. The soft impacts pivot here between potential additional care time for patients and the deskilling of physicians in providing informed opinions. Thus, looking at soft impacts and mediations reveals the moral complexity of introducing ML systems into medical practice, encompassing both individual and practice-based concerns.

#### The responsibility of patients

ML shapes the relationship between health care professionals and patients and ties patients to certain images and responsibilities [8, 11]. Because ML systems rely on quantifiable data sets, they lend themselves to a construal of patients as data points and threaten to relegate patient experiences and relational issues to the background [5, 47, pp. 16–21]. In doing so, ML implicitly shapes the responsibility of patients: for health care professionals to take contextual factors neglected by ML into account, patients need to be very vocal in bringing their illness experiences and relational factors to the fore.

The idea that it is in patients’ best interest to proactively describe their situations to health care professionals fits with the logic of patient empowerment that often accompanies the introduction of new technologies in medical practice [48, 49]. Such empowerment discourses frame technologies as a means of independently managing medical conditions using the supposed objectivity of health data, without the need to rely on the subjective judgments of health care providers. Software designers and developers directly engage in such discourses when touting their work as having the potential to empower patients “to make accurate and interpretable data-driven clinical decisions” [50]. But is the increased responsibility on patients to communicate the context and specifics of their medical history to medical professionals indeed a form of empowerment?

From the perspective of TMT, the answer depends on how ML mediates the relationship between health care provider and patient. If ML threatens to relegate the personal context of patients to the background, then the extent to which providers will take patient narratives into account within the diagnostic process remains to be seen. Mutatis mutandis, the supposed objectivity of ML-aided diagnoses might also serve to silence patients’ experiences of illness, especially since patients already often perceive medical professionals as attempting to downplay these experiences [51, 52]. Accordingly, ML potentially reconstitutes the dynamics of the relationships between health care professionals and patients, imposing specific forms of responsibility that each party might experience as a burden: while health care professionals must take responsibility for what ML does *not* reveal and independently augment ML with personal and contextual information about their patients, patients must be able to articulate such information explicitly to their health care providers and communicate candidly and vocally about how they experience medical problems.

#### The responsibility of developers

In considering the existential concerns for health care professionals and patients discussed above, it becomes apparent that many anticipated issues are conditioned by the affordances and implementation choices embedded within ML systems. Thus developers are brought into the spotlight as indirect agents in diagnostic processes.

Unsurprisingly, considerable attention has been paid in the literature to the roles and responsibility of design in ML systems used in health care. One issue often raised relates to how developers can help to make ML models more transparent. Recently, concerns about opacity have been addressed in regional regulatory initiatives promoting the ethical development of AI (e.g., in Europe and China) by stipulating a principle of explainability, providing that ML systems be made “auditable, comprehensible and intelligible by human beings at varying levels of comprehension and expertise” [53, p. 10] or “traceable, auditable and accountable” [54]. However, as Federico Cabitza and colleagues suggest, the task of familiarizing medical professionals with the decision-making logic of ML systems is challenging because it requires finding a balance between the accuracy of the model and its interpretability [5, p. 518].

Diagnoses made by ML systems tend to be presented as categorical interpretations of a medical condition—potentially at the expense of rich clinical observations that take into account the contexts of particular patients and can “measure and compare intangible goods so that they can be weighed and pitted against one another” when making a diagnosis and developing a treatment plan [43, p. 59]. Accordingly, there is a worry that ML systems implicitly moralize the diagnostic process. This concern is intensified by the visual authoritative bias that people have regarding technologies [55], especially when dealing with recommender systems that bypass contextual information and have not been developed by or in consultation with physicians or other trained health care providers [6].

To counter this implicit feature of ML systems, Israni and Verghese confer a distinct moral role to developers—to “help clinicians deliver better and more humanistic care,” specifically by enhancing their “capacity to love, to have empathy, to care and express caring, to be generous, to be brave in advocating for others, to do no harm, and to work for the greater good and advocate for justice” [46]. Gordana Dermody and Roschelle Fritz point to participatory design as a step in the right direction, providing an example of a framework that integrates the knowledge and values of medical stakeholders, including clinical nurses, in developing ML systems [56].

Overall, the existential concerns related to design center around the responsibility that developers have to allow health care professionals and patients both to constructively manage the inevitable mediating effect of ML systems on their perceptions and actions and to deal with its soft impacts on their roles, identities, and practices. The emergence of the moral principle of explainability—codified in initial attempts at regulating the practice of AI development [53, 54]—mirrors these concerns, offering new ethical signposts to navigate and shedding fresh light on existing ones (e.g., the principles of diversity and inclusion regarding ML/AI bias). Thus, even though responsibility is implied in the process of creating new technologies, the implementation of ML for diagnostic purposes invites developers to revisit and reinterpret their role in view of the unique features of ML systems.

### Legal concerns

The existential concerns discussed above might not only reshape the roles of health care providers, patients, and developers, but also spur changes in the interpretation of medical legislation. Given that ML and other applied AI create ambiguity as to who is responsible for medical diagnoses, some suggest that the role of ML systems in medical decision-making should be legally accounted for [37, p. 42], which may require rethinking the nature of responsibility and accountability in medical contexts as well as the legal status of ML systems.

Earlier, we discussed how health care professionals’ responsibilities and duties are reconfigured by ML systems. Legally speaking, this reconfiguration involves reforming the categories of medical malpractice, vicarious liability, and product liability, as well as the ancillary duties of health care providers. Many of these ancillary duties traditionally fall under the rubric of soft law—presenting self-regulating obligations and codes of conduct that are not always legally enforceable but are core to good practice. In the context of health care, such duties might include the duty to warn or protect, to ensure informed consent, to avoid conflicts of interest, and so forth. With the introduction of AI systems, some argue that certain ancillary duties, such as the duty of loyalty and the duty to warn, might be reallocated to hard law, interpreted as legal obligations related to disclosure of information [42, pp. 384–385].

The legal questions that ML systems give rise to concern not only health care providers, but also the systems themselves. Generally, health care technologies, including software, fall under medical device regulations that maintain standards for their safety, quality, access, use, and disposal [42]. However, traditional laws may not be equipped to cover the scope of ML’s contribution to medical decision-making, especially as used in AI that exhibits a certain level of autonomy, that has the ability to continually learn and update prediction models, and that obfuscates the logic behind suggested output [57, 58]. As Jason Chung and Amanda Zink note in the case of Watson, “We have a hodgepodge of theories of recovery for injuries due to medical treatment—primarily medical malpractice, vicarious liability, and products liability—but Watson doesn’t fit neatly into any of these categories” [43, p. 63].

Some suggest that medical AI may spur legal reform, helping to optimize existing regulations and adapt them to the capabilities of new technologies [42, p. 384]. Others offer more radical and philosophically challenging solutions, such as granting a degree of legal personhood to AI in health care to account for its active role in medical decision-making [43, 59]. Chung and Zink suggest, that in terms of liability, AI systems should be treated as medical students operating under the supervision of a principal attending physician [43]. Classifying medical AI systems as legal persons would allow ML diagnostic models to fit within existing medical malpractice schemes in the event of misdiagnosis. Nonetheless, as the authors acknowledge, in reality, “medical students are virtually never pursued for medical malpractice as a result of diagnostic error given the overriding responsibility of the attending physician” [43, p. 73]. Even if the analogy the authors propose is brought to bear, then, the burden of responsibility, accountability, and liability would in all likelihood still disproportionately lie on health care providers.

Whatever the legal solutions end up being, this discussion illustrates the challenges ML systems and other AI pose to the regulation of health care. AI mediates the meaning of medical responsibility and accountability, as well as reforms existing ancillary duties, possibly shifting some from soft to hard law. An anticipated soft impact of medical AI involves reconsidering the legal status of such technologies as agents in medical practice, potentially bringing them closer to personhood and further blurring the distinction between human and machine. While the issues and questions presented above are broadly relevant within the American and European legal traditions, it is important to remember that legal systems vary from country to country, each existing and developing within a unique historical and cultural context. As a result, specific soft impacts and mediations in the legal realm must be analyzed in the light of particular local practices and environments. However, the general point stands: the discussed mediations and soft impacts are characteristic of anticipated and debated changes to the legal infrastructure—one that will have to be adapted or revisioned to incorporate emergent technologies with the potential for autonomy and agency in medical diagnosis.

## Evaluating machine learning through the lenses of technomoral change and technological mediation theory

What is revealed about the complementarity of TMC and TMT by applying them to analyze ML in medical diagnosis? In this section, we discuss this question in terms of (1) the extent to which these approaches serve to highlight the qualitative moral impact of ML and (2) the potential for a combination of TMC and TMT to contribute to participatory engagement in the context of medicine.

### Qualitative moral impacts: beyond risks and harms

In the introductory section, we suggested that qualitative moral impacts are underrepresented in the ethical discourse on ML, and advanced TMC and TMT as two approaches that can help to compensate for this underrepresentation. Guided by these two perspectives, we then distilled epistemic, existential, and legal concerns expressed in the literature on ML and AI systems in health care and analyze the anticipated moral impacts on health care providers, patients, developers, policymakers, and other stakeholders. Generalizing from this analysis, one could say that the qualitative moral impacts of ML and AI in medical practice have been tacitly interpreted in the dominant ethical discourse according to the doctrine of double effect, such that introduction of these technologies is justified by virtue of the *intended* quantifiable benefits to the health care system and in spite of any *foreseen* qualitative changes to medical practice, relations between patients and health care professionals, normative categories, and so forth. Even if the potential qualitative moral impacts are acknowledged, they are treated as vague and decontextualized, and priority is given to bold statistical predictions about touted improvements to accuracy, efficiency, safety, and the like. However, we believe that the complex web of intertwined epistemic, existential, and legal concerns brought into focus by TMC and TMT warrants closer attention when considering the introduction of ML systems in medical diagnostics. It is precisely because these foreseen concerns are not intended that they should be discussed on a par with intended benefits in the decision-making process.

Following Swierstra and te Molder [10], our focus has been on the soft impacts of introducing ML in medical diagnosis, thereby attending to how ML modifies “our relations, our values, our norms, our aspirations, our situation definitions, the meanings we attach to the world” [60, p. 203]. Such soft impacts sit in contrast to the hard impacts of health technologies—that is, their rational, objective, and measurable effects on patient outcomes, such as the likelihood of misdiagnosis. In addition, following Verbeek [18], we have shown how the use of ML in medical diagnosis mediates the morality of medical practice, and specifically how it mediates the notion of what counts as good health care. Ultimately, not only does ML work to constitute situated experiences, understandings, perceptions, and actions, but its influence on moral conduct also mediates the moral frameworks used to evaluate diagnostic practice.

The lenses of TMC and TMT allow anticipated qualitative moral impacts to be discerned and examined because the approaches are designed to target concerns that usually fly under the radar of decision-makers by virtue of their supposedly nonrepresentational and fuzzy nature. As Swierstra and colleagues maintain, just because such concerns do not fit within traditional hard-impact-oriented frameworks of decision-making, such as risk assessment or cost–benefit analysis, does not mean that they are less important [10, 28, 60]. Understanding shifts in health care professionals’ perception of their profession may seem trivial compared to immediately tangible calculations of potential cost reductions brought by ML systems. However, if these shifts cause health care professionals to perform under the implicit pressure to comply with suggestions made by ML systems and lead them to doubt their own diagnostic abilities, then they may present a real risk of harm to patients and health care professionals alike.

Even if decision-making about new technologies initially skews toward cost–benefit logic, direct causality, and numerical appraisal, there could still be a way to incorporate qualitative concerns. However, before such concerns can be incorporated, they first have to be identified and analyzed. To that end, both TMC and TMT can help to foreground the qualitative moral impacts of technologies like ML—impacts that threaten to disappear from view due to the dominant focus on hard impacts in ethical discussions about algorithms and AI. Accordingly, the analysis conducted in this paper can complement and augment ongoing efforts to promote an informed and critical introduction of ML systems into health care.

### Participatory engagement and co-designing the implementation of machine learning

As observed above, the discourse on ML in health care is largely concerned with ways of eliminating human bias in machine diagnosis, presenting such bias as a technical obstacle to the proper functioning of ML systems. However important the issue of bias is, the myopia of such discussions ultimately fails to represent or appreciate the full nature and scope of medical expertise. As elucidated by TMC and TMT, ML changes medical expertise by construing it as a matter of formal pattern recognition, thereby prioritizing a certain model of medical diagnosis over the tacit, not always propositionally expressible expertise that health care providers draw on when diagnosing patients and presenting treatment options. If the development of ML systems continues to take place predominantly in technological domains, then less visible but no less crucial aspects of medical diagnosis risk being overlooked. To increase the visibility of nontechnical concerns when developing such systems for health care, contributors to an AI-focused publication by the US National Academy of Medicine suggest participatory engagement as a form of codesign practice [1, p. 161].

Ensuring that development teams for ML diagnostic systems are inclusive and diverse—in terms of gender, age, race, culture, socioeconomic background, and so forth—should enable the qualitative moral impacts to be foregrounded early on in the design process [1, pp. 22–23]. The identification of moral impacts should not be a purely theoretical endeavor undertaken only within the confines of the academic literature, but should first and foremost have a practical benefit to users of these systems—most importantly, to health care providers, patients, and software developers. Establishing the potential soft impacts and mediations of ML should help the relevant actors understand their own roles and their relation to one another. Participatory engagement throughout different stages of the developmental lifecycle of ML medical systems will help various actors to come to a shared understanding of stakeholder-specific concerns. The process of codesign equips participants with a repertoire for expressing how they are affected by the introduction of ML and for engaging in a dialogue about whether these effects are desirable. Access to such a shared repertoire can also facilitate participatory methods like focus groups and stakeholder meetings by mitigating the conceptual barriers to productive dialogue. In the absence of a definitive solution to the problem of how ML can be introduced responsibly into medical practice in consideration of its qualitative moral impacts, it becomes clear that the morality of ML should be regarded as collective and emergent—taking a form that is locally negotiated by and among users.

Of course, we are not so naïve as to think that a discussion of soft impacts and mediations will straightforwardly change the way technological innovations are introduced into medical practice. It is hard to ignore the power imbalances in this regard, as government agencies, insurance companies, and large health care corporations rarely consider the input from medical professionals or patient associations prior to imposing new technologies upon them. While it is important to reflect on the politics of technological innovation in health care, it is equally important to address the way new technologies are appropriated in clinical practice. We suggest that analyses inspired by TMC and TMT can do just that, contributing to the critical, informed, bottom-up development and appropriation of ML for use in specific health care contexts—tailored to particular practices, doctors, and patients. However modest, such a reflective and deliberative practice-focused approach can empower medical professionals and patients alike to critically appropriate ML without waiting for changes in the top-down dynamics of technological innovation.

TMC and TMT can each contribute to the formation of a reflective community by equipping actors with relevant conceptual frameworks for thinking through their relationships with medical technologies. While TMC primarily investigates how technologies shape routines that are often not directly accessible for conscious reflection, TMT focuses on how actors develop new ways of understanding themselves as moral subjects and how technologies mediate the notion of what constitutes the good life or good medicine. Given that it involves various levels of individual and institutional perspectives, participatory engagement lends itself to a combination between the TMC and TMT approaches. A combination affords a more comprehensive vision of how participatory engagement should be organized than each of the approaches can offer on its own. This combination can help actors in developing a vocabulary for engaging in what Swierstra calls technomoral learning, namely the willingness to “investigate, when problems pressure us into doing so, all thinkable solutions—be they primarily technological or be they primarily moral in character” [60, p. 216]. Showing that the use of ML in medical practice creates new responsibilities for health care professionals, patients, and developers can help when thinking about the kind of skills or expertise each of these stakeholders might need to facilitate its introduction.

## Conclusion

In this paper, we have shown how the approaches of technomoral change and technological mediation theory can be used to elucidate which qualitative moral impacts are anticipated when ML is introduced into the process of medical diagnosis. We analyzed the concerns raised by ML systems on three different levels: epistemic, existential, and legal. In doing so, we showed (1) how ML shapes the production of good clinical knowledge and what health care professionals’ responsibilities are in this process, (2) how ML affects the way that health care professionals, patients, and developers understand themselves as responsible actors in relation to one another, and (3) how ML calls for potential revisions to legal regulations in order to provide for its evolving role in medical diagnosis. Instead of offering clear-cut solutions to the foreseen moral impacts of ML, our analysis suggests that these impacts may be most appropriately conceptualized not as finite problems but as uncertainties, which take emergent form through collective association with the technology and thus to which no definitive answer can be proposed in advance of its introduction.

Acknowledging the uncertainties involved when anticipating the qualitative moral impacts of ML might also help to offset the epistemic stratification of participants in medical encounters, according to which practitioners are considered unilateral information providers and patients are reduced to mere information receptacles. After all, in terms of qualitative moral impacts, health care providers and patients are in the same boat, and they must work out on an equal basis whether specific impacts of ML are ultimately desirable or not. A focus on the soft impacts or technological mediations that ML gives rise to might help in this regard because it establishes a vocabulary for articulating how ML assigns new responsibilities to health care providers, patients, and developers and for identifying which forms of expertise might be needed to facilitate its introduction.

One possible direction for future research on the qualitative moral impacts of ML in medical diagnosis would be to examine whether the moral concerns that are (implicitly) expressed in the literature align with what health care professionals and patients themselves anticipate. The concerns identified in this paper can be used as a blueprint for discussing diagnostic ML systems in focus groups or stakeholder meetings, as well as for venturing into more specific contexts of application, such as mental health care. To that end, the two approaches presented in this paper invite a view of technologies as active albeit subtle parties in medical decision-making processes. Acknowledging and trying to account for the qualitative moral impacts of ML systems can give depth and substance to the current debates and help users to make informed decisions about the introduction of these technologies in health care.
